# Guidelines for managing and using the digital phenotypes of pluripotent stem cell lines

**DOI:** 10.1016/j.stemcr.2024.08.009

**Published:** 2024-09-26

**Authors:** Christine A. Wells, Anke Guhr, Amos Bairoch, Ying Chen, Mengqi Hu, Peter Löser, Tenneille E. Ludwig, Nancy Mah, Sabine C. Mueller, Andrea E.M. Seiler Wulczyn, Stefanie Seltmann, Bella Rossbach, Andreas Kurtz

**Affiliations:** 1 Stem Cell Systems, Department of Anatomy and Physiology, Medical, Dentistry and Health Sciences, University of Melbourne, Parkville, VIC 3010, Australia; 2 Robert Koch Institute, 13353 Berlin, Germany; 3 University of Geneva and SIB Swiss Institute of Bioinformatics, CMU, 1 Rue Michel Servet, 1211 Geneva, Switzerland; 4 Fraunhofer Institute for Biomedical Engineering (IBMT), Joseph-von-Fraunhofer Weg 1, 66280 Sulzbach, Germany; 5 WiCell Research Institute, Madison, WI, USA; 6 Berlin Institute of Health Center for Regenerative Therapies at Charité, Berlin, Germany

## Abstract

Each pluripotent stem cell line has a physical entity as well as a digital phenotype, but linking the two unambiguously is confounded by poor naming practices and assumed knowledge. Registration gives each line a unique and persistent identifier that links to phenotypic data generated over the lifetime of that line. Registration is a key recommendation of the 2023 ISSCR Standards for the use of human stem cells in research. Here we consider how community adoption of stem cell line registration could facilitate the establishment of integrated digital phenotypes of specific human pluripotent stem cell (hPSC) lines.

## Graphical abstract

**Figure:**
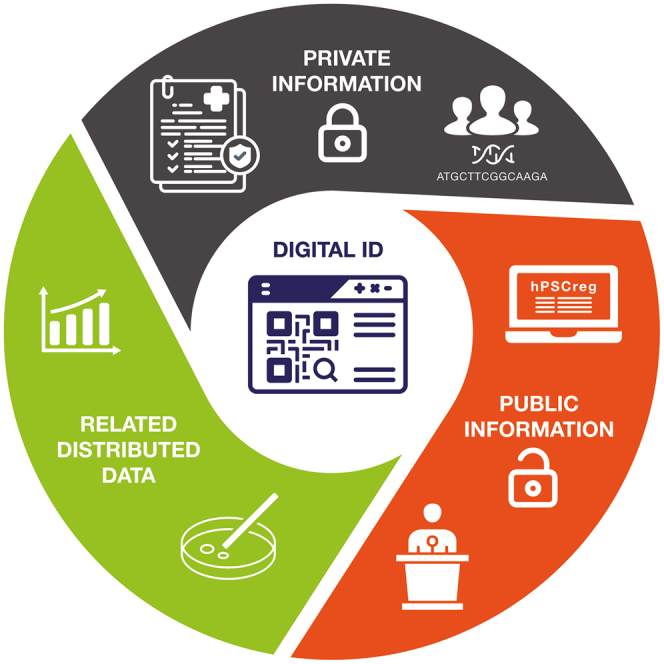

## Teaser

In this article, Kurtz, Wells, and colleagues consider how the registration of stem cell lines will contribute to the development of new digital phenotypes of stem cells and their derived materials and further provide an important evidence base for the equitable uptake of stem cell technologies.

## Quality data practices are critical for research integrity

The ability of human pluripotent stem cells (hPSCs) to differentiate into many cell types of the human body and to form organoids and blastoids, makes hPSCs excellent *in vitro* systems for modeling simplified aspects of the original human donor (Vandana et al., 2023). Individual hPSC lines are edited, distributed, and differentiated for clinical translation and commercial applications, including drug safety and efficacy evaluations. This includes screening for genotypes susceptible to drug-induced long QT syndrome in human induced pluripotent stem cell (hiPSC)-derived cardiomyocytes (Bellin et al., 2013), producing preclinical models such as the engraftment of hiPSC-derived reporter lines for Parkinson’s disease (Moriarty et al., 2022) and developing haplotype-matched hiPSCs for clinical trials (Yoshida et al., 2023). Large and genotypically diverse collections of hiPSCs are being generated to support cohort-scale research into disease such as Parkinson’s disease (Bressan et al., 2023) and amyotrophic lateral sclerosis (Workman et al., 2023), among many others. However, information about these efforts can be siloed in project-specific databases, restricting data sharing between even related disease groups. Equally important, verifying the ethical provenance associated with the generation and downstream use of hPSCs, including clinical or commercial applications, is hampered when related data about a line is siloed.

Research is a collaborative endeavor such that sharing and re-purposing cell lines and their associated datasets is necessary for publication and downstream research translation. The scale and diversity of hPSC research necessitates that the community agrees to robust data management approaches. This starts with the unambiguous identification, traceability, and verification of hPSC lines, and associated data that persist beyond single projects (see Box 1 for glossary of terms). The recent standards developed by ISSCR to facilitate reproducible stem cell research clearly demonstrates that it is not sufficient to derive a generalized phenotype from a handful of lines without clearly defining the experimental variables underpinning the model (ISSCR Steering Committee and Working Task Force on Standards for Human Stem Cell Use in Research, 2023; Ludwig et al., 2023). This includes details about cell line derivation, characterization, and culture conditions. Failure to implement data initiatives that support transparent and traceable cell line provenance risks compromising the reproductibility of results, and more generally, impacts trust in emerging fields of hPSC-derived therapies.

Box 1 **Glossary of terms**

**Table:** 

**Genetic identity** is synonymous with the *biological identity* of a line. It is derived from the donor and is standardly used to authenticate cells and cell lines derived from the donor. For example, STR profiles or equivalent, haplotyping, SNP arrays, or whole-genome sequencing can be used to validate the authenticity of biological material by matching the genetic identity of the donor and the sample. **Biological phenotype** describes the characteristic phenotypes of a cell line. For hPSC, the inherent biological phenotypes include pluripotency, cell morphology, and accompanying transcriptomic or metabolic features, and may further be extended to include phenotypes derived from hPSC-derived cells, tissues, and organoids. While the *biological identity* of a line remains largely stable, *biological phenotypes* may vary under different experimental conditions. **Digital identifier** is a unique and persistent code that is issued by an authoritative *cell line registry*, such as hPSCreg (hPSC registry), Biosamples (data repositories for NCBI, DDBJ, EBI), and Cellosaurus (encyclopedia of cell lines used in biomedical research). The Cellosaurus identifiers act as Research Resource Identifiers (RRIDs), the use of which is now mandatory in the method section of many journals. Digital identifiers are machine-readable database codes that support the use of human-readable cell line names, including colloquial name changes. E.g., the embryonic stem cell line colloquially known as either H9 or WA09 has the persistent digital identifier WAe009-A, which also links modifications created on that line back to the parental source. **Digital phenotype** encompasses the body of data that is linked to a *digital identifier*. A digital phenotype requires a data file, usually a matrix of numbers, and a metadata file that explains the experimental setup, relevant sample information, and includes information about how the data file was generated. It is essential to link phenotypic datasets associated with the cell line, using a *digital identifier* that has been issued and maintained by a centralized and authoritative *cell line registry*. **Digital provenance** describes the ethical provenance of cell line derivation, allowing for fundamental information about what a donor has consented to, and should include information from ethical review boards such as informed consent documentation provided to the donor. Data access and usage conditions associated with a line should be summarized in machine-readable profiles, such as those proposed by Common Conditions of Use Elements (Del Carmen Sanchez Gonzalez et al., 2023) and Digital Use Conditions (Jeanson et al., 2023). **Cell line registry** refers to a freely accessible online resource that reviews cell line data before assigning a systematic, unique, and persistent *digital identifier* to the cell line. A registry allows for sharing key information about the derivation of a stem cell line with search functions on the online registry or federated data sharing with other data resources. The organizations that manage the registry have robust governance practices to manage the cell line data in the registry and are recognized by the stem cell community as authoritative sources of cell line information. **Cell line catalog** refers to the online listings of banked cell lines that are available for distribution, for example, through not-for-profit cell repositories or commercial entities. These catalogs may link a banked line to its *digital phenotype* via its registered *digital identifier*.

## Creating persistent and interoperable cell line identifiers

Even though many individual laboratories work on a relatively small number of hPSC lines, the problem of finding information about those lines is difficult when thousands of seemingly related hPSC lines exist in the public domain. User-generated cell line names are often neither unique, stable, nor persistent. Changes to the annotation of lines within a lab may make sense—for example, when operator-derived or representing a clonal event in the history of the line—but the rationale behind the naming is rarely transparent to others, and naming is not coordinated between labs. Some examples of commonly used names include some variation of hiPSC1/2/3 and clone 1/2/3, some variation of the parental cell and a number (e.g., BJ1-iPSC1 and PBMC-iPSC1), or a disease abbreviation and number (e.g., PMF1/2/4). These are all examples found in the online knowledge-based Cellosaurus.

Cellosaurus (Bairoch, 2018) aims to describe all cell lines used in biomedical research. To populate the knowledge resource, it manually curates literature and patent reports, integrates information provided by cell line collections and companies developing/distributing cell lines, and increasingly from direct submissions by research groups and consortia. 20% of the entries in Cellosaurus are pluripotent stem cell lines, in total 30,984 hPSC lines out of 112,768 human cell lines (152,231 of any species as of January 2024). Each cell line is represented in the Cellosaurus by a single entry to which a unique, stable identifier is assigned. In late 2016, Cellosaurus joined the Resource Identification Initiative (RII) (Bandrowski et al., 2015), which aims to promote research resource identification, discovery, and reuse.

A critical goal of the RII is the widespread adoption of Research Resource Identifiers (RRIDs) to promote citations of biological resources such as antibodies or other biological reagents, including cell lines. Attribution of this material should be possible anywhere that references their generation or use and is exemplified in, but not limited to, peer reviewed publications. The RII has put in place a Resource Identification Portal (https://scicrunch.org/resources) to collate these RRIDs. Cellosaurus is the cell line resource for the RII, such that cell line RRIDs are also the Cellosaurus accession number. Stem cell registries share the identifiers that they generate with RII resources, including Cellosaurus. These initiatives provide a mechanism to assign a stable and unique digital identifier that can be used in parallel with the line’s common use name. A registered digital identifier for a cell line is analogous to a digital object identifier (DOI) for a paper acting as an adjunct to the human-readable citation, or the way the gene symbol links the many synonyms associated with a gene product to a common origin (Seal et al., 2023). However, while a DOI is automatically assigned to a publication, registration of lines requires active participation of the stem cell community.

Stem cell registries provide more than just a persistent identifier; they curate information about the source and ethical provenance of a cell line. Early stem cell registries were established by public funders in response to ethical concerns about using public resources to fund embryo research. For example, human embryonic stem cell (hESC) registries were established by the US National Institutes of Health (NIH) (National Institutes of Health, 2024; Loring and Rao, 2006) and the European Commission (hESCreg, the forerunner of the current hPSCreg; Borstlap et al., 2008) to allow the use of vetted hESC lines in publicly funded research projects (Luong et al., 2008). One of the key functions of these early hESC registries was thus the assessment, validation, and certification of specific cell lines, their ethical provenance, and/or their biological characteristics. Once reprogramming technologies became widely available, hESCreg co-opted its original goals to re-brand itself as hPSCreg, a resource-agnostic cell line registry for hESC and hiPSC lines (Seltmann et al., 2016; Mah et al., 2020; Kurtz et al., 2022). Though primarily a European initiative, cell lines from all over the world can be registered by the cell line generator at hPSCreg, which names and identifies hPSC lines using a broadly supported standard nomenclature (Kurtz et al., 2018). hPSCreg identifiers are interoperable with Cellosaurus and therefore part of the RRID network.

Through efforts like Cellosaurus and registries like hPSCreg, there are thousands of hPSC lines already captured in some digital format. Inevitably, there are also pockets of hPSC lines hidden from the global community and which cannot be unambiguously identified due to variable naming conventions and the lack of peer-reviewed publication of these lines, for example, cohorts of lines deposited in cell banks, which have not yet been cited by researchers. Even for highly regulated hESC lines, we found that only a moderate proportion of the more than 2,300 original hESC lines established and published so far made its way into international stem cell registries such as the human pluripotent stem cell registry (hPSCreg; Seltmann et al., 2016) or the US NIH Human Embryonic Stem Cell Registry (National Institutes of Health, 2024; Loring and Rao, 2006) (Table 1). From the Australian stem cell community, there are 302 hPSC lines lodged for unique identifiers issued by hPSCreg, but only 114 of these have the minimal information about their derivation needed to be fully validated for this registry. There are over 1,300 Australian hiPSC lines, some published with accompanying omics data (Daniszewski et al., 2022; Wilson et al., 2022), which are not yet captured in any registry. These data suggest that not only are Australian lines under-represented in registries, but that they are also largely hidden from RRID generators such as Cellosaurus. Tracing the provenance of these lines relies entirely on the details provided at the time of publication.

**Table 1:** Registration of hESC lines in hPSCreg and in the NIH Human Embryonic Stem Cell Registry as of 15th March 2024

Country	No. of hESC lines				
	hESC numbers	Number of validated hPSC lines in hPSCreg	Percentage	NIH	Percentage
US	873	261	29.9	262	30.0
China	463	68	14.7	0	0.0
Israel	172	24	14.0	124	72.1
UK	146	92	63.0	34	23.3
Korea	130	34	26.2	0	0.0
Sweden	123	94	76.4	6	4.9
Australia	115	22	19.1	66	57.4
Belgium	71	38	53.5	0	0.0
Spain	43	37	86.0	0	0.0
Iran	40	10	25.0	0	0.0
Denmark	33	30	90.9	0	0.0
France	32	22	68.8	0	0.0
Czech Republic	23	15	65.2	0	0.0
Russia	20	3	15.0	0	0.0
India	19	9	47.4	2	10.5
Japan	18	9	50.0	1	5.6
Turkey	18	18	100.0	0	0.0
Finland	17	14	82.4	0	0.0
Singapore	15	14	93.3	6	40.0
Taiwan	15	8	53.3	0	0.0
Thailand	14	3	21.4	0	0.0
Brazil	6	1	16.7	0	0.0
Switzerland	6	4	66.7	0	0.0
Canada	4	2	50.0	2	50.0
Netherlands	4	4	100.0	0	0.0
Mexico	2	2	100.0	0	0.0
Colombia	1	0	0.0	0	0.0
**Total**	**2,423**	**838**	**34.6**	**503**	**20.8**

Shown are the numbers of publicly known original hESC lines, the numbers of registered cell lines, and the percentage of hESC lines registered in the registries indicated. Publicly, hESCs were identified by regular inspection of the relevant literature as well as by searches in publicly accessible databases. For details, see the study by Guhr and colleagues (Guhr et al., 2018). Please note that sublines derived from an original hESC line (e.g., genetically modified hESC clones) are not included.

## Registries promote the interoperability of data from different domains

In the world of scientific data management, being findable, accessible, interoperable, and reproducible, or FAIR, addresses the need for scientific reproducibility by using formats that allow verification of the original publication’s findings (Wilkinson et al., 2016). Publication alone is not an appropriately FAIR mechanism for sharing information about a cell line, since publication results are not necessarily sufficiently annotated with rich metadata, which would render them machine-readable and machine-actionable, a key FAIR requirement. Once a stable digital identifier has been established by registering the cell line, the cell line ID becomes the “handle” for all data associated to that line, including biological properties as well as ethical and legal conditions of use. It is important to stress that all existing public or laboratory-assigned non-public names remain attached to the cell line as synonyms, and therefore cell lines remain findable by their non-standard names. Thus, all data assigned to this unique digital identifier reproducibly form part of the cell lines’ digital phenotype (Table 2).

**Table 2:** The digital phenotype of hPSC or its derivatives has three components: a description of its generation, characterization, and authentication

Generation	Characterization	Identity/authenticity Original line or Genetically modified line.	
Donor information (e.g., source cell type, phenotypic features, genomic data)	Expression of informative proteins/RNA and other omic profiles (“stem cell markers,” transcription profiles)	Genome features (e.g., karyotype, genome sequences)	
		Authenticity (STR fingerprints, SNP/HLA profiles)	
Reprogramming method	Potency (e.g., differentiation into three germ layers)		Modified genomic feature (e.g., gene, region, SNP)
Cultivation details			Verification and characterization of the modification (e.g., sequencing, protein expression)
Microbiology/virology/sterility			
If modified: modification method			

This information accompanies a line’s digital identity and provenance (see glossary for definitions).

There is no single resource that has a monopoly on cell line data. Each resource has its own niche, and rather than re-inventing the wheel, it is more practical to link and share data between resources. Beyond community decisions on “what” data should be recorded (ISSCR Steering Committee and Working Task Force on Standards for Human Stem Cell Use in Research, 2023; Ludwig et al., 2023), the next question is “how” to record the data, such that registries can connect to multiple resources (e.g., other registries, cell banks, catalogs, directories, repositories, citation databases, etc.) in a way that they understand each other and easily interpret and share the data. The use of metadata standards is essential for this interoperability.

Metadata formalizes information about a cell line or experiment, and stem cell registries have developed schemas to capture relevant metadata about pluripotent stem cell lines not otherwise reviewed by other RRID providers. These include details about the way the lines were made, including the source tissue or cell used for reprogramming, data that evidence the pluripotent characteristics of the line, and the conditions under which the hPSC lines are cultured. Metadata can be based on established ontologies and taxonomies, or on stakeholder-defined naming conventions. For example, ontologies for diseases allow the harmonization of multiple terms used to describe the same disorder (e.g., diabetes mellitus, T2D, diabetes type 2 etc.), removing ambiguities in a cell’s digital phenotype. Using ontologies also helps researchers find related disorders, which may be useful for comparisons of cell phenotypes for drug screening, or functional gene testing. Following metadata standards permits a more systematic and machine-driven comparison of the reported cell line characteristics between different lines. Consequently, the biological properties of cell lines can be directly compared when ontologies are used for cell type, derivation method, or methods used to assess pluripotency or differentiation characteristics. In this way, the use of common data vocabularies by registries broadens the applicability of cell line data, while helping to simultaneously improve existing and develop new standards relevant to applied pluripotent stem cell biology.

## Enablers of registration

There are relatively few studies that have evaluated mechanisms that promote cell line registration; however, from these few, effective levers for registration include community articulation and adoption of reporting standards (Ludwig et al., 2023), journal adoption of ISSCR guidelines (ISSCR Steering Committee and Working Task Force on Standards for Human Stem Cell Use in Research, 2023), and the requirements of some regulators or funders such as in the EU, Korean, USA, and UK to register ESC and, in some instances, induced pluripotent stem cell (iPSC) lines generated within their jurisdiction (Kington, 2009; Lee et al., 2011; Aran et al., 2020; O'Shea and Abranches, 2020; European Commission, 2021; Official Journal of the European Union, 2021).

Institutional support for registration, including funding for infrastructure development and ongoing maintenance, is essential, and withdrawal of these has led to the loss of some key international registries (Luong et al., 2008; Kim et al., 2017). Availability of ongoing funding for database projects is a particular barrier for global adoption outside of Japan, US, and EU. These registries increase the value of the resources, as they align with ongoing efforts of global research communities and funders toward FAIR data models. There is an argument for local development of infrastructure, because these also offer hubs for training, as well as the development and implementation of local standards, languages, and culturally appropriate metadata models. Establishing such resources in global settings offer opportunities for Western data generators and cell line custodians to adopt culturally appropriate data models, as well as develop protocols for data exchange where access may require custodial or community review (Riedel et al., 2020; Carroll et al., 2021).

## Equity and global representation

An important aspect of hPSC registries is to document the ethical provenance of cell lines, their availability to be shared, and where relevant, any conditions associated with their use. Where hPSC lines can be shared, it is important for researchers to be easily able to discover information such as the information provided to a donor at the time a line was generated, including the informed consent documentation. This puts the onus on potential users of these lines to decide whether their planned application is consistent with the derived consent (Table 2). Understanding the relationships between founding and modified lines, the ethically compliant provenance of these lines, and any restrictions in sharing or secondary use of a line or its associated data also facilitates good governance of those lines across the stem cell community (Isasi et al., 2014, 2019).

**Figure 1:**
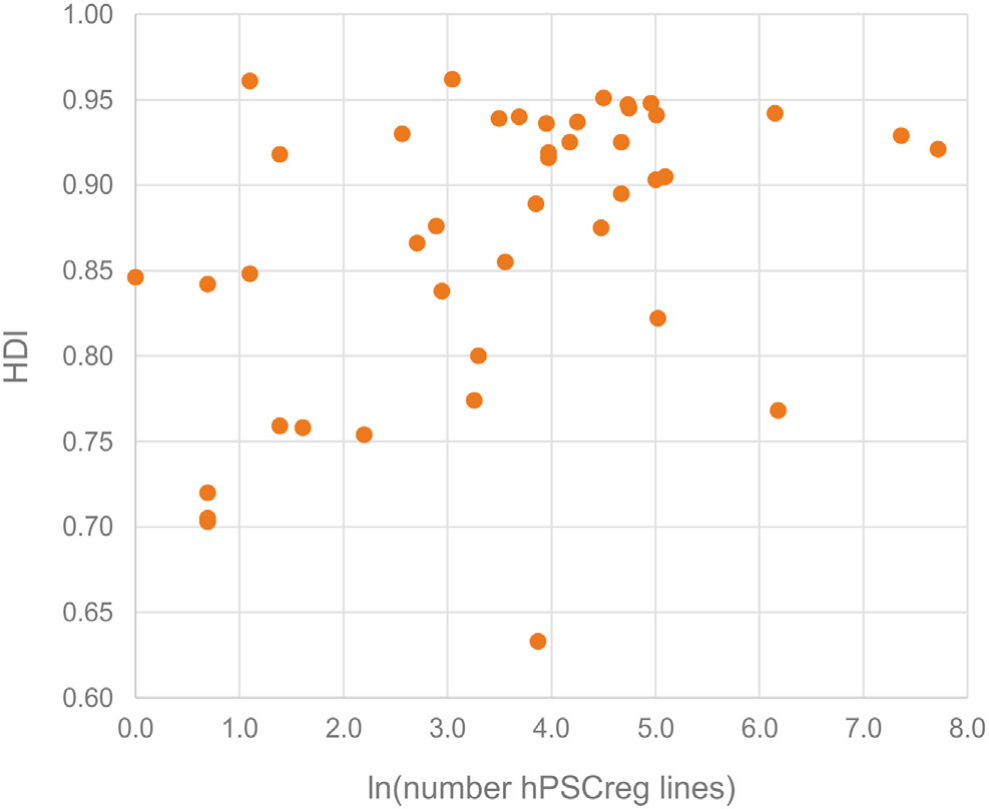
Wealthy countries generate the highest numbers of hPSC lines Countries with very high Human Development Index (HDI) generate a high proportion of cell lines worldwide. Countries are classified as: (1) very high human development: HDI>0.800, (2) high human development: 0.700 < HDI <0.800, and (3) medium human development: 0.550 < HDI <0.700. Number of registered cell lines in hPSCreg is represented by the natural log of all submitted cell lines (6,825 lines as of March 17, 2024) per country. The Human Development Index (2021) was sourced from https://hdr.undp.org/data-center/human-development-index#/indicies/HDI.

These issues of consent become even more important when considering social, economic, and/or geographical disparities in stem cell line availability. Equity in stem cell derivation and use must consider three aspects of potential donor vulnerability: socio-economic status, disease status, and geographical ancestry. Most of the hPSC lines in cell line resources originate from wealthy industrialized countries, the diseases common to industrialized nations, and subsequently the predominant geographical ancestries present in these nations. Estimates from registered lines in hPSCreg (international) show that >90% of hPSC lines are generated in countries with “very high human development” (Figure 1), as measured by the Human Development Index, which is a metric of average achievement in three key dimensions: life expectation, education, and standard of living (United Nations Development Programme, 2024). The geographical ancestries of the donors represented by the cell lines in hPSCreg could be broadly inferred from the ethnicity data provided in 3,344 (28%) hPSC lines in hPSCreg (Table 3). Markedly, there is a dearth of cell lines from first nations peoples who reside in the Americas, Australia, and Africa. Although Table 3 is only based on cell lines registered at hPSCreg, we can anticipate that inclusion of geographically isolated populations will be rare, or at best incidental to the collection of lines with active hiPSC programs. Furthermore, 42.5% in hPSCreg (2,879) originate from donors without a diagnosed disease. The diseases represented by the remaining lines in hPSCreg show that approximately one-third of lines (902) encompass common non-communicable diseases found in industrialized countries, such as Alzheimer disease (291 lines), Parkinson disease (223 lines), diabetes mellitus (242 lines), and macular degeneration (146 lines). Cell lines from donors with rare diseases account for approximately one-fifth of registered lines, but these resources remain scarce because, by their nature, these samples are much more difficult to obtain and access is often restricted. Others have reported on the lack of representation in hPSC lines and further suggest that targeted increases in global genetic representation must be accompanied by uplift in the stem cell research capabilities across poorly represented nations (Ghosh et al., 2022). This gap in diversity increases the drive to preserve precious lines and make these available as far as ethically possible. Failure to build equitable resources will not only hinder research that may benefit these groups but also represent a looming equity gap if these groups are consequently excluded from new stem cell therapies. Globally accessible registries may provide a platform to find and access such lines.

**Table 3:** Geographical ancestries of cell lines registered in hPSCreg

Geographical ancestry region	Number of hPSC lines in hPSCreg
Europe	1,674
East Asia	301
Africa	90
South Asia	62
Middle East	40
Multiple	16
Americas	4
Oceania	0
Unknown	4,638
Grand total	6,825

Note that as ethnicity is a social construct, geographical ancestry regions were manually assigned to cell lines, based on the availability of data collected by the “ethnicity” free text field in hPSCreg. Broad assignments are as follows: Africa: African American; Black, any country in Africa; East Asia: China, Japan, Korea, Thailand, Taiwan; Europe: White, Caucasian, European, Ashkenazi, Hispanic, any country in Europe; Middle East: Arab, Jewish, Persian, Saudi, Yemenite; Americas: Alaska, Mexico; South Asia: India, Asia, Sri Lanka, Bangladesh, Pakistan, Afghan; Multiple: more than one description of ethnicity was provided; Oceania: (hPSCreg did not have any lines in this group) Borneo, New Guinea Highlands, Fiji, Polynesia, Indigenous populations of Australia and New Zealand; Unknown: free text ethnicity data could not be mapped to broad geographical regions (ambiguous or missing data).

One important consideration when creating stem cell lines from groups who have suffered from past medical exploitation is ensuring that the CARE principles (Collective benefit, Authority to control, Responsibility, and Ethics) are at play—a commitment to community benefit, including creating capacity to translate research outcomes within that community, by community members, and in partnership with community priorities (Carroll et al., 2021). This may also mean protected access models of data and lines such that the governance of these lines is also in partnership with the donors.

An essential feature of such a resource is trustworthiness with regards to data handling, data assessment, their safety and security, as well as sustainability. Specifically, when data are trusted to a registry, validated, cleared, and quality certificates are issued, it is necessary to establish methods to minimize data breach, the unintentional or intentional amassing of wrong or outdated data, and to have clear and transparent guidelines on the applied standards and methods for data management. Registries must work closely with stakeholders, who may also serve as independent auditors of the resource. Because registries are open for public access, they provide opportunity for the public to follow scientific developments in the field, including clinical application of hPSC-derived cell products. In addition to offering an independent audience to verify the data in the registry, registries can link to comprehensive resources providing regulatory and ethical guidance, training, and dissemination materials, thereby pointing to regulatory, ethical, and societal implications and frameworks related to stem cell work.

## Future facing – Building composite phenotypes

Creating platforms for unambiguously capturing information about hPSC lines is a powerful engine for data mining. Future use cases for registries include linking their current functions to store and share data, with an ability to map new data to a specific line, using a persistently linked cell line identifier. Such a registry platform with its data models, standard vocabularies, and information encoding enables the application of advanced data analysis methods such as machine learning to reveal the power of stem cells’ digital phenotypes toward e.g., assessing the safety of hPSC-based drug screens or other applications. Emerging examples include the Stemformatics.org platform, which integrates RNA sequencing (RNA-seq) from many laboratories to form transcriptional atlases used to benchmark iPSC-derived cells against their *in vivo* counterparts, which is particularly useful in a field where different differentiation protocols or cell line propensities may impact on the quality of the final, derived tissue (Choi et al., 2023).

Relationships between cell lines are also important to capture but can be obscured if not deliberately captured in a registry setting. Genetically modified hPSC lines can be such valuable tools that they may be more widely distributed and adopted in the research community than the founder line, which increases the risk that the link between subclone and its origin is lost. For example, the knowledge gained from a specific subclone might reveal new biological insights when correlated with information generated by another subclone from the same parental line; without the biological provenance of these lines, relevant data cannot be integrated as part of a larger, comprehensive analysis. Opportunities will thus be missed toward building a broader stem cell network based on linked data and deciphering the individual versus common contributions.

The molecular phenotypes associated with an hPSC line depend on the intersection of individual donor genetics with each derived cell lineage (Kilpinen et al., 2017). Increasingly, these molecular phenotypes rely on high-dimensional molecular data such as RNA-seq, metabolic profiling by mass spectrometry, or other emerging and data-rich technologies (Cahan et al., 2021). While journals may ask authors to share some data (e.g., RNA-seq) via a public repository, poor-quality metadata means that it can be difficult to find and assess data from an individual line. In reviewing data for the Stemformatics atlas from publications that utilize more than one cell line, and especially in studies involving hESC and hiPSC for the same experiments, we observe the tendency toward aggregating data rather than representing outcomes from a specific hESC or hiPSC line. This makes sense when seeking to summarize generalizable observations from the study; however, when this summarization spills over into digital housekeeping for the study, it obscures individual biological properties of hPSC lines that may impact on the behavior of the model and so are key for reproducible and trustworthy research. Up to 30% of published stem cell data fail curation by community resources such as the Stemformatics team because poor-quality sample metadata means it is difficult to identify which cell lines were used (Choi et al., 2019, 2023).

Equally important is the realization that data are most valuable when it can be reused—benchmarking new cell models, discovering new aspects of the biology through reanalysis, and supporting modern machine learning toolkits for biological pattern recognition—all these require data to be in a FAIR and structured format. Most journals ask that data generated from each contributing line is traceable in the primary data accompanying each study, but this in turn requires a simple method to match lines to studies, and within a study to individual figures or tables. We argue that using a registry to unambiguously assign a unique digital identifier to a line is the only way to support the development of reporting systems that unambiguously link lines to reported data. These FAIR ideals are underpinned by common data management practices that are not yet commonly adopted by the stem cell community. Registries are a relevant mechanism to implement such practices. Moreover, only by such a mechanism can traceable and reproducible generalizations be made across studies, and potentially interesting outliers be identified.

The fast development of science and the growing number of cell lines, models, and applications form an emerging “datadom”—a complex digital phenotype comprising a diversity of metadata models, data access, and control formats. Much of this extended data is best served in specialized databases, which can be linked to the associated hPSC line via its digital identifier. As an example, hPSCreg records a BioSamples identifier (Courtot et al., 2019) for each registered hPSC line, which is indispensable to link deposited “-omic” data in three major biological data repositories (EGA-Europe, DDJB-Japan, and NCBI- US) to the cell lines in hPSCreg. Such data may be protected because of requirements in different jurisdictions to protect donor privacy. By maintaining the digital identities of cell lines, registries can facilitate controlled access to sensitive datasets by managing the links between public information and the data vaults protecting sensitive (personal) data such as whole-genome DNA sequencing. Similarly, publications and the data within publications should be tagged with registered cell line identifiers, ideally as machine-readable metadata associated to the publication, or provided in a structured format within the main text of the publication. This is important since most original experimental data on the characteristics and potentials of a specific hPSC lines are provided in a multitude of research papers. The approach of linking hPSC lines to publications reporting experimental work involving the use of the respective hPSC line and deposited “-omic” data has already been employed by both hPSCreg and Cellosaurus. However, the consistent use of cell line identifiers and metadata in machine-readable versions of journal publications needs to be embraced by publishers and the stem cell community in order to maximize data re-use.

## Potential for registries to support adult stem cell models, including organoids

While the scale and fragmentation of hPSC data collections necessitates urgent action on data standards, the stem cell community is also adopting primary cell models that can be expanded, banked, and shared, such as gut, skin, cornea, and other epithelial tissues. Data provenance and research integrity are even more acutely impacted when the lines themselves have a limited shelf life or restricted sharing options, as the community cannot rely on accessing the primary material to repeat scientific claims. Registration of organoid collections is supported by the assignment of unique digital identifiers such as RRID, but this does not associate key quality metrics with the collection. Registration of these primary cell assets would be beneficial in terms of building a legacy digital phenotype of cell lines, and of properties shared by tissue type, biopsy site, and other donor categories such as age, health status, or genotype. A unique and stable digital identifier provides a secure way to anonymously summarize protected donor data with experimental readouts. Whether suitable registries exist is a matter for further consultation with the adult tissue community.

## Conclusion

Registration of hPSC lines, either via dedicated registries such as hPSCreg or via curated services such as Cellosaurus, initially sought to capture key information about how a line was generated, characterized, and its ethical provenance documented. New opportunities for accumulating digital phenotypes associated with hPSC lines build an even stronger imperative to unambiguously identify hPSC lines, their progeny, and associated distributed data. Reference databases are clearly needed to serve as anchors for the classification of cells for basic, preclinical and clinical research and other applications. The ability to compare between banked and lab-siloed cells is however complicated by the absence of commonly accepted and scientifically sound reference datasets, as well as generally accepted reference cell lines. Therefore, a definite need presents itself for the development and visualization of standard criteria for the description of cell lines in order to facilitate their use in research and therapeutic purposes, which would in turn provide datasets for reference purposes. In addition to creating reference datasets for cell lines, a common metadata resource could integrate tools for data exchange and comparison, to enable the user to search for data among all available cell lines in all dedicated cell resources. By annotating cell lines with biological identifiers such as authenticating short tandem repeat (STR) profiles, comparisons against all available authenticated cell lines can be performed (Robin et al., 2020). The ISSCR standards cope with these challenges and the rising challenge of digital identities and the need for registration of cell lines to make data management fit for research reproducibility and emerging data-driven applications. Utilizing the experience of the established projects like hPSCreg and Cellosaurus, which already exchange and harmonize information on cell lines in their databases, will forward the cross-integration of frameworks and build the common data portal in addressing the problems previously outlined.

## Acknowledgments

The authors acknowledge and thank Daniel Goscha, WiCell Technologies, for assistance with the graphical abstract. Funding to C.A.W.: MRFF2008807 from the Australian Medical Research Future Fund and from the NCRIS Australia fund via Phenomics Australia. Funding to A.K.: 10.13039/100018693EU Horizon Europe GA 101074135.

## Author contributions

Conceptualization, C.A.W., T.E.L., and A.K.; data curation, A.B., A.G., A.E.M.S.W., P.L., M.H., N.M., B.R., S.S., and Y.C.; writing – original draft, A.B., A.K., P.L., N.M., and C.A.W.; writing – review and editing, A.B., M.H., T.E.L., S.C.M., A.K., and N.M.; supervision, P.L., A.K., and C.A.W.; project administration and funding acquisition, C.A.W. and A.K.

## Declaration of interests

A.K. is the coordinator of the Horizon Europe-funded registry hPSCreg, and C.A.W. is head of the Phenomics Australia-funded Australian stem cell registry. T.E.L. is a co-inventor and receives a share of royalties on various hPSC media and culture-related patents currently held and licensed by the Wisconsin Alumni Research Foundation.
